# Socio-demographic, institutional and governance factors influencing adaptive capacity of smallholder irrigators in Zimbabwe

**DOI:** 10.1371/journal.pone.0273648

**Published:** 2022-08-29

**Authors:** Liboster Mwadzingeni, Raymond Mugandani, Paramu L. Mafongoya

**Affiliations:** 1 School of Agriculture, Earth and Environmental Sciences, University of KwaZulu-Natal, Scottsville, Pietermaritzburg, South Africa; 2 Department of Land and Water Resources, Faculty of Agriculture, Environment and Natural Resources Management, Midlands State University, Gweru, Zimbabwe; Universitat Autonoma de Barcelona, SPAIN

## Abstract

The provision of resilience and adaptation to climate change to smallholder irrigation communities is a critical component in implementing common pool resource management. Institutions in many smallholder irrigation schemes in developing countries are diverse and have potential to contribute to building climate resilience and improving livelihoods of smallholder irrigator. Human behaviour, institutional capacity and culture play important roles in shaping adaptive capacity of communities to climate change. Although much is known about how these contribute to this adaptive capacity, research focusing on their interaction is limited. In order to close this the gap, this study seeks to explore how socio-demographic, governance and institutional factors influence adaptive capacity in Exchange, Insukamini and Ruchanyu irrigation schemes. Questionnaire-based interviews, group discussions and key informant interviews were used for data collection. Adaptive capacity calculated using the livelihood vulnerability model was used as the dependent variable for this study. Ordinary least square regression was used to assess socio-demographic, institutional and governance factors influencing adaptive capacity in the smallholder irrigation scheme. The study reveals that adaptive capacity is influenced by age, gender, education, land tenure security, irrigation committee satisfaction, cooperatives, and interaction of factors. The link between age and gender were negatively moderated by awareness of irrigation policies, access to credit and land tenure security. Assessing factors influencing adaptive capacity help to improve the livelihoods of scheme farmers in the face of climate change.

## 1. Introduction

Adaptive capacity is crucial to ensure that systems, institutions, humans, and organisms can innovate and adapt more quickly to climate change and variability [1]. Building adaptive capacity has become more imperative in global policies documents such as Agenda 2030 [2], the Paris Agreement [3] and, growing interdisciplinary research. Further, following the Intergovernmental Panel on Climate Change (IPCC) Third Assessment Report (TAR) [4], there has been a rapid growth of studies on adaptive capacity, methodologies and, matrices (indices, proxies and scores) [1]. Adaptive capacity is defined by IPCC assessment report AR5 [5] as, “the ability of the systems, institutions, human, and other organisations to adjust to potential damage, to take advantage of opportunities, or to respond to consequences.” Assessment of adaptive capacity has grown exponentially across scales [6] sectors and geographies since its recognition as a critical component of vulnerability and resilience [1, 7]. Adaptive capacity studies are dominant in developed nations but limited in developing nations [1].

Smallholder irrigation schemes (SISs) are promoted to increase the adaptive capacity of communities in marginal environments. These SISs are common-pool resources (CPRs) and their success is dependent on the robustness of self-governing institutions and their capacity to sustainably manage the productive resource as conditions changes [8, 9]. The strength of the institutions governing socio-ecological systems like irrigation schemes is important to most smallholder farmers’ livelihoods and food security [10]. Yet, socio-ecological systems have numerous complex variables that interact and affect how they operate at multiple levels [11, 12].

Previous studies have shown that the institutions in the SISs should be effective in collective decision-making, allocation of limited water resources, infrastructure maintenance, and conflict resolution [9, 13]. Institutions thrive on building and sustaining cooperation in CPR system through satisfying both short-term self-interests and long-term group goals [14] in an evolving ecological, socio-economic and political environment shaping its emergence, evolving and operation [13]. SISs are run by Irrigation Management Committees (IMCs), which are mainly governed by customary laws and water use rights that authorise scheme farmers to collectively manage water use [9]. Nevertheless, environmental change and increased water scarcity have added an extra burden on these functional socio-ecological systems that have shown mixed performances [8]. The relationship between CPR management and adaptation calls for integrating climate and socio-ecological system management policies [9, 15] in SISs. Institutions can constrain or enable adaptation [16]; hence institutional analysis is essential in adapting socio-ecological systems to climatic change.

As an integral part of smallholder irrigation schemes, governance is vital for addressing climate change-related challenges in these socio-ecological systems. According to institutional theory, governance involves decision-making and resource utilization to alter the conditions of the society [17]. Several studies have shown that governance shapes adaptive capacity [18–23]. Governance is essential for climate change adaptation in smallholder agriculture [24, 25] and smallholder irrigation farming [26]. Recent studies show that improvement of system governance enhanced climate change adaptation [27–30].

Socio-demographic factors are essential for climate change adaptation in smallholder irrigation schemes [31]. A recent study in Nepal shows that socio-demographic factors are among the factors which influence adaptive capacity [31]. According to Alemayehu and Bewket [32], socio-demographic factors affect farmers’ choice of coping and adaptation strategies to climate change. Socio-demographic characteristics, including household size, education, age, and gender, are basic farmer characteristics that influence their potential to adapt [31].

This study was inspired by Ostrom [12] through her institutional analysis framework developed to understand and overcome uncertainties and complex social dilemmas. Reflections across different CPR systems show the success and failure of Ostrom principles [13, 33]. This is not surprising given the myriad and complex relationship between institutions and adaptation in SISs. Globally, the findings from publications on the roles of institutions on adaptation in SISs have produced contrasting results under different settings [13]. This shows that previous successful institutional actions may not be effective in a different context, creating a knowledge gap on patterns and dynamics of institutional changes in diverse socio-ecological and political contexts [13]. Therefore, given the heterogeneity of the farming systems worldwide, especially in southern Africa, more evidence-based studies are required to explore the role of institutions in adaptation in SISs. This scientific inquiry is critical given that in the absence of unambiguous local knowledge, the role of institutions in adaptation in SISs would largely remain a challenge. The CPR nature of SISs highlights the need to understand the relation between institutions and adaptation to climate change. SISs in southern Africa and especially in Zimbabwe may differ from similar CPR worldwide based on their biophysical, cultural and political context, challenging understanding of the performance of their institutions on adaptation based on existing studies.

In Zimbabwe, Vision 2030 foretells a completely different economy for the country. In agriculture, a low-hanging fruit sector for Vision 2030, a sensible problem is climate-proofing crop yields in smallholder farming systems given their vulnerability to climate change. By developing the targeted 350 000 ha for irrigation during the Zimbabwe National Development Strategy 1 (2020–2025), the country aims to increase agricultural output, particularly in smallholder farmers. IMCs running SISs are key to climate-proofing crop yields. Thus, policy strategies are required to strengthen institutions for reducing sensitivity while building resilience to climate change.

Based on the literature on the role of institutions on adaptation in the smallholder irrigation systems [8, 15, 34–38], we make the following conjectures: Institution influence the adaptive capacity of smallholder irrigation systems; Institutional elements such as scheme rules, governmental and private organization support, water sharing and scheme maintenance, IMCs and participation of scheme member influence adaptive capacity.

Zimbabwean context is suitable for studying the relationship between institutions and adaptation in SISs due to multiple reasons. First; Irrigation schemes in the country have a linear relationship with higher food security and income that is compromised by drought-related challenges and limited investment in infrastructure maintenance [39]. Secondly, a majority of the SISs have collapsed [40], while some are performing poorly [39]. Thirdly, Zimbabwe lies in southern Africa, where poor farmer participation, lack of access to markets, weak institutions, limited political will, poor scheme design, and technical factors are highlighted as some of the critical factors undermining the performance of SISs [39, 41, 42]. Fourth, the government of Zimbabwe and its partners in irrigation development, including the International Fund for Agricultural Development (IFAD) and the Smallholder Irrigation Revitalization Programme (SIRP), continue to invest in new SISs and rehabilitate existing ones. Lastly, through the recently availed Irrigation Policy, the Government of Zimbabwe intends to improve water use efficiency, improve access to finance, inputs, markets, overcome governance challenges, and improve policy and regulation environment in irrigation schemes. Thus, strong institutions in SISs are required to achieve these objectives.

Our study focuses on two social factors that are of paramount importance in climate change adaptation: age and gender. Vulnerability to climate change differs widely at the household level up to the national, reflecting the probable influence of age and gender, among other factors, through multiple pathways [43].

Despite contributing about 80% of the total food produced in Africa, women face an assortment of challenges [44]. The high prevalence of gender inequalities among developing nations, particularly in Africa, is a cause of concern considering the threats posed by climate change and limited adaptive capacities, particularly in women [45]. Gender shapes the outcome of climate change for smallholder and subsistence farmers [46]. Poor women and female-headed households are disproportionately on the receiving end of increased environmental degradation and have fewer resources to cope with and adapt to climate change [45, 46]. For instance, rural areas have increased walking distances to fetch water and firewood. Adaptation to climate change is not gender neutral due to the tendency of existing wide gender inequalities [47]. Gender plays a crucial role in climate change response since women are affected differently and their lack of power to influence decision-making than men. Thus, understanding the interaction of gender and institutional factors requires special attention when mainstreaming gender in adaptive capacity actions.

Age determines vulnerability to climate change and other environmental shocks, as the impacts of climate change are differentiated along the age lines. Further, the impacts of climate change are age-differentiated and reflect personal experiences. Climate action will not succeed without the participation and engagement of old, as 10% of the world population is currently over the age of 60 years, while 20% will be over the age of 60 by 2050. The old farmers have lived experiences in the past and present climatic conditions, positively influencing their adaptation to climate change [48]. The experience of old farmers make them risk averse compared to young farmers [48]. However, age positively impacts climate change adaptation, as aged farmers are more enlightened about climate change and various livelihood options to adapt. While literature is awash with the role of socio-demographic, governance, and institutional factors on adaptive capacity, there is less insights on theorization on interaction among variables. Meanwhile, there is need for research on adaptive capacity to shift focus from the relationship between two variables towards establishing how some putative causal variables influence the outcome through answering “*how*” and “*when*” questions [49]. This helps to deepen our understanding of the phenomenon through uncovering and describing the contingencies of mechanisms [49]. Conditional process analysis was widely used in empirical literature across many disciplines: psychology [50–52]; public health [53]; sociology [54]; management [55]; biology [56]; and communication [57]. However, its use in climate change vulnerability and adaptation is at an infancy stage. Therefore, this study seeks to quantify and test the hypothesis by which socio-economic, governance and institutional transmit their effects on adaptive capacity based on ex-ante expectations in smallholder irrigation schemes in Zimbabwe.

## 2. Methodology

### 2.1 Study area

This study was conducted in Midlands Province of Zimbabwe. It progressed from the identification of three research sites (Exchange, Insukamini and Ruchanyu Irrigation schemes). The selection of the study sites was based on the diversity of their characteristics, as shown in Table 1.

**Table 1:** Characteristics of Exchange, Insukamini and Ruchanyu irrigation schemes.

Variable	Scheme		
	Exchange	Insukamini	Ruchanyu
Year constructed	From 1973 and from 1985	1988	1980s
Location	Zhombe communal area, Silobela	Lower Gweru community	Shurugwi
Land area	168.8 ha	41 ha	27 ha
Distance from town	60km from Kwekwe 80km from Gweru	41 km from Gweru	29 km from Shurugwi
Agroecological zone	4	4	3
Number of households	982	125	85
Source of water	Exchange dam	Insukamini dam	River
Water delivery	Concrete canals	Concrete canals	Sprinkler
Management system	Consultative and democratic	Consultative and democratic	Consultative and democratic
Rainfall	450–650 mm	600–800 mm	650–850 mm
Temperature	26°C	16°C	16°C
Soils	Clay loam	Sand loam and clay loam	Sand loam
Main crops and average yields	Maize (7 t/ha) Beans (1 t/ha in winter and 1.2 t/ha in summer)	Maize (4.4 t/ha) Beans (1.9 t/ha) Onions, cabbages, tomatoes, wheat, peas, garlic and rape	Maize

Data in the table was sourced from [58–62]

### 2.2 Data collection

Questionnaire surveys were undertaken to collect data in Exchange, Insukamini and Ruchanyu irrigation schemes. A pilot study was done to test the suitability of the questionnaire for this study. Questionnaire interviews were used to collect socio-economic, institutional and governance data from the head, middle and tail sections of the schemes.

This study used stratified random sampling to identify respondents based on statistical representativeness of the sample. Random sampling is a probabilistic sampling method brought to the fore by Bowley [63]. A statistically significant sample of 317 households (192 from Exchange irrigation scheme, 88 from Insukamini irrigation scheme and 37 from Ruchanyu irrigation scheme) was selected for the study (P ≤ 5%) as shown in Table 2. The formula below was used to determine the sample size of this study:

$$n=\frac{N}{Ne^{2}}$$ (1)

Where *n*–sample size, N–population and *e*–confidence interval

**Table 2:** Sampling design.

Irrigation Scheme	Population Size	Sample Size
Exchange	982	192
Insukamini	125	88
Ruchanyu	85	37
**Total**	**1 192**	**317**

### 2.3 Ethical statement

As guided and approved by the Institutional Review Board of the University of KwaZulu-Natal (HSSREC/00003196/2021), ethical requirements were followed. Participants signed a consent form, were informed that they could stop the interviews at any time, that there were no consequences for non-participation and that data would be treated confidentially.

### 2.4 Data analysis

Firstly, adaptive capacity was computed from raw data set using weighted-balance and integrated approach. Weighted balance and integrated approach adapted after Hahn et al [64] was used to calculate adaptive capacity. Major components of adaptive capacity (socio-demographic factors, livelihood strategies and social networks) consist of subcomponents, each contributing equally to the index and given equal weighting. The weighted balance and integrated approach is mainly used in calculating Livelihood Vulnerability Index (LVI) and Climate Vulnerability Index [65]. The use of weighted balance and integrated approach is recently increasing due to the rising need to analyse vulnerability to climate-related disasters [65].

Factors such as awareness of irrigation policies, access to credit, and satisfaction with tenure security were used to condition the link between gender and age of the household head with adaptive capacity. Meanwhile, the link between independent factors, interactions and adaptive capacity was estimated by linear regression analysis at the same time. Hayes [49] postulate that analysis of interactions can be done using various models, hence, for this study we used linear regression model. Ordinary least-square (OLS), one of the familiar statistical techniques in the social sciences used to predict the values of continuous response variables in multivariate analysis [66], was applied. Variance inflation factor (VIF) less than 2.96 less than a cut-off point of 10 [67], shows absence of potential multicollinearity. The Breusch-Pagan/Cook-Weisberg test show absence of heteroskedasticity (Chi2(14) = 11.02; p = 0.808). OLS satisfies the need of this study due to its ability to provide the best estimates with continuous and coded categorical variables [66]. Recently, OLS was used to assess the effects of climate change adaptation on livelihood vulnerability in Ghana [68]. Linearity of regression coefficients, absence of serial correlation, predictors are uncorrelated with coefficients, absence of multicollinearity and normality of residuals are the limitations met to have the best OLS estimates from this study.

Eq (2) was used to standardize specific components.

$$Index_{sv}=\frac{S_{v}-S_{min}}{S_{max}-S_{min}}$$ (2)

Where S_v_−original subcomponent value; S_min_ and S_mux_−minimum and maximum value of the subcomponent, respectively.

An average of each subcomponent was calculated using Eq (3)

$$M_{vj}=\frac{\sum_{i=1}^{n}Index_{svi}}{n}$$ (3)

Where M_vj_−value of major component j for area v; Index_svi_−subcomponent value indexed by *i* of major component M_j_; n–number of subcomponents in major component Mj.

$$IndexAdaCap=\frac{W_{SDF}SDF+W_{LS}LS+W_{SN}SN}{W_{SDF}+W_{LS}+W_{SN}}$$ (4)

Where *IndexAdaCap*–adaptation capacity index; W_SDF_, W_LS_, W_SN_−weight for socio-demographic factors (SDF, livelihood strategies (LS and social network (SN), respectively.

An OLS regression analysis was performed on vulnerability indices of Adaptive Capacity to assess governance and institutional factors influencing the adaptive capacity of scheme communities. OLS regression was performed with Adaptive Capacity as a dependent variable, while socio-demographic factors, governance and institutional variables.

$$Y_{i}=a+b_{1}X_{1}+b_{2}X_{2}+\ldots +b_{m}b_{m}+c$$ (5)

Where *Y*_*i*_−the dependent variable; *X*_1_, *X*_2_,…,*X*_*m*_−the independent variables; *a*–constant; *b*_1_, *b*_2_,…,*b*_*m*_−multiple regression coefficients.

## 3. Results

### 3.1 Socio-demographic characteristics

Table 3 below shows socio-demographic characteristics of households in Exchange, Insukamini and Ruchanyu Irrigation Scheme. Based on this study, Ruchanyu Irrigation Scheme has the most male respondents and Insukamini have the least (P ≤ 0.01). Among the three schemes, most of the respondents in Ruchanyu were significantly married than respondents in Ruchanyu and Exchange, given that over nine-tenth of the respondents were married (P ≤ 0.01). Although more scheme farmers where married, the majority of the female household heads (53.8%) were not married, as shown in Table 3. It was observed that Exchange is dominated by aging farmers (average age of 56 years) compared to Ruchanyu and Insukamini Irrigation schemes. Data from the questionnaire survey revealed that respondents from Insukamini Irrigation scheme acquired the highest level of formal education (10 years).

**Table 3:** Socio-demographic variables.

Variable	Frequency	Mean	Percentage	Significant level	Standard deviation
Gander (Male)					
Exchange	122		63.5	***	
Insukamini	52		59.1		
Ruchanyu	70.3		70.3		
Marital Status (Married)					
Exchange	135		70.3	***	
Insukamini	63		71.6		
Ruchanyu	35		94.6		
Gender and marital status of household heads (Married)					
Male			89.5	***	
Female			46.2		
Age					
Exchange		56.24			12.78
Insukamini		52.30			
Ruchanyu		53.14			
Years of formal education					
Exchange		8.53			3.14
Insukamini		10.02			
Ruchanyu		8.90			
Size of household					
Exchange		4.52			2.22
Insukamini		5.85			
Ruchanyu		7.42			
Number of years in farming					
Exchange		31.24			14.38
Insukamini		20.45			
Ruchanyu		20.15			
Years in irrigation farming					
Exchange		23.59			12.74
Insukamini		11.23			
Ruchanyu		9.36			

Note: *** indicate significance level at 1%.

In comparison, those in Exchange irrigation scheme attain the least educational level (8.5 years). In respect of household size, respondents in Ruchanyu Irrigation Scheme have the largest household size (average of 7.42 members) while the respondents in Exchange Irrigation Scheme had the least (average of 4.52) as shown in Table 3. Respondents in Exchange Irrigation Scheme are more experienced farmers (average years in the farming of 31.24 years), while those in Ruchanyu had the least experience in farming (average years of farming of 20.15 years). Respondents in Exchange Irrigation Scheme are more experienced in irrigation farming (average of 23.59 years), while those in Ruchanyu Irrigation Scheme are last experienced (average of 9.36 years) (Table 3).

### 3.2 Governance and institutional factors

Across the three irrigation schemes in this study, there is a significant difference in satisfaction of participation of local institutional actors in the schemes (P ≤ 0.01) as shown in Table 4.

**Table 4:** Institutional factors of Exchange, Insukamini and Ruchanyu irrigation schemes.

Scheme	Strongly disagree	Disagree	Neutral	Agree	Strongly agree	*X*^2^ Significance level
**Effectiveness of traditional leaders in irrigation farming**						
Exchange	1.6	5.7	30.2	53.6	8.9	***
Insukamini	6.8	6.8	72.7	13.6	0	
Ruchanyu	5.6	11.1	44.4	22.2	16.7	
**Cooperatives**						
Exchange	19.3	11.5	34.9	31.3	3.1	***
Insukamini	50.9	23.6	21.8	3.6	0	
Ruchanyu	0	25.0	18.8	12.5	43.8	
**NGO and PVT Organizations**						
Exchange	0.5	0	0	28.1	71.4	***
Insukamini	0	6	57.8	36.1	0	
Ruchanyu	13.3	40	10	16.7	20	
**Academic Institution**						
Exchange	0	0.5	7.3	57.3	34.9	***
Insukamini	1.6	18.8	65.6	12.5	1.6	
Ruchanyu	0	11.1	14.8	66.7	7.4	
**Irrigation Committee**						
Exchange	3.1	1.6	10.4	43.2	41.7	***
Insukamini	1.1	2.3	2.3	62.5	31.8	
Ruchanyu	3.4	10.3	6.9	55.2	24.1	
**Community/Fellow Farmers**						
Exchange	0.5	0	1.6	40.6	57.3	***
Insukamini	0	14.7	5.9	63.2	16.2	
Ruchanyu	3.3	10	16.7	30.0	40.0	
**GVT Agencies (Extension Workers)**						
Exchange	0	0	2.1	21.6	76.30	***
Insukamini	0	0	2.4	48.2	49.4	
Ruchanyu	0	3.7	7.4	44.4	44.4	

Note: *** indicate significance level at 1%.

For the three schemes in this study, there is a significant difference in the participation of scheme farmers in irrigation water scheduling and electing/removing members (P ≤ 0.01) (Table 5).

**Table 5:** Governance factors of Exchange, Insukamini and Ruchanyu irrigation scheme.

	Never	Sometimes	Always	Significancy
**Participate in irrigation water scheduling**				
Exchange	8.9	42.7	48.4	***
Insukamini	24.1	5.7	70.1	
Ruchanyu	3.1	9.4	84.4	
**Participate in electing / Removing committee members**				
Exchange	11.5	39.6	49.0	***
Insukamini	1.1	13.6	85.2	
Ruchanyu	0	21.2	78.8	
**Access to credit**				
Exchange	19.8	55.7	24.5	***
Insukamini	18.2	12.5	69.3	
Ruchanyu	0.0	15.2	84.8	

Note: *** indicate significance level at 1%.

### 3.3 Factors affecting adaptive capacity

A zero-order correlation was applied to determine the magnitude of the correlation of socio-demographic, institutional and governance factors with adaptive capacity. Table 6 shows the descriptive statistics and correlation among variables, including factors that significantly and positively or negatively correlate with Adaptive capacity.

**Table 6:** Descriptive statistics and correlation among variables.

	HH_Age	HH_Gender	HH_Edu	Tenur	Tradit	Coop	Pvt_NGOs	Acade	Irri_comm	Gvt_agenc	Polic	Credit	Ada_Cap
HH_Age	1.00												
HH_Gender	0.06	1.00											
HH_Edu	-0.54**	0.20**	1.00										
Tenur	0.12*	0.10	-0.04	1.00									
Tradit	0.12*	0.04	-0.15*	0.261**	1.00								
Coop	0.11	0.27**	-0.06	0.10	0.02	1.00							
Pvt_NGOs	0.07	0.03	-0.04	0.34**	0.20**	0.22**	1.00						
Acade	0.02	0.05	-0.04	0.38**	0.21**	0.14*	0.44**	1.00					
Irri_comm	-0.02	-0.07	0.035	-0.06	-0.03	-0.26**	0.04	0.19**	1.00				
Gvt_agenc	0.05	-0.06	0.04	-0.06	0.25**	-0.13*	0.28**	0.28**	0.43**	1.00			
Polic	-0.05	0.09	0.09	0.30**	0.40**	0.04	0.29**	0.48**	-0.11	0.21**	1.00		
Credit	0.05	-0.08	0.04	-0.22**	-0.03	-0.34**	-0.30**	-0.19**	0.30**	0.14*	-0.02	1.00	
Ada_Cap	0.35**	0.51*	0.16**	0.02	0.16**	0.33**	0.22**	0.10	0.06	0.03	-1.46**	0.01	1.00
SD	12.78	0.48	3.14	0.38	0.84	1.22	1.00	0.84	0.87	0.56	0.56	0.74	0.10
Min	18.00	0.00	0.00	0.00	0.00	0.00	0.00	0.00	0.00	2.00	0.00	0.00	0.46
Max	87.00	1.00	17.00	1.00	5.00	5.00	5.00	5.00	5.00	5.00	1.00	2.00	0.99

Note: N = 317

*—p ≤ 0.05;

**—p ≤ 0.01

The age of the household head is a proxy of farming experience which positively influences adaptation to climate change [48]. Older adults have understanding, skills and philosophies developed by societies over interaction with natural surroundings and indigenous knowledge, which help them cope, adapt and impress diverse livelihood options to changing climate [43]. Older people have past and present environmental knowledge helping them to detect, understand and predict environmental change [43]. Agrarian communities in the Global South are marked with gendered inequalities where males have dominant abilities to hold and manage land, access credit and access to development programs distinctively designed for men [69]. Education reflects the capacity to generate, absorb and process new information, assess response options and frame or reframe problems [43]. Education enables effective adaptation to climate change by preparing people for complex adaptive decision-making [70]. Land tenure security strongly incentivizes farmers to invest in climate change adaptation [71]. Traditional leaders in Zimbabwe have been useful in the governance of people in rural communities and can play a supportive role in climate change adaptation by enforcing traditional strategies for combating the negative effects of climate change [72]. Local farmers’ cooperatives, academic institutions, the private sector and non-governmental organizations provide an opportunity to implement climate-smart agricultural technologies, which sustainably increase yields under climate change [73]. Irrigation committees provide ways to enhance water-use efficiency and address water paucity across schemes [73]. Enabling policies and institutional mechanisms facilitate the scaling up of adaptation throughout the agri-food systems [73]. Access credit promotes investment in agriculture for climate change adaptation [74].

A moderated regression on adaptive capacity was performed to test for the hypothetical moderation effect of socio-demographic and institutional factors. HH_Gender, Polic, HH_Age, Credit, HH_Edu, Schedule, and Tenure were mean-centered to moderate them and link their interplay with adaptive capacity. According to Aiken, West [67], mean-centering reduces multicollinearity and simplifies results interpretation. The interplay was calculated by multiplying socio-demographic factors and institutional factors. The moderation results of socio-demographic and institutional factors are shown in Table 7. Regression equation for adaptive capacity was found to be significant (F = 14.13; p < 0.001). The results in Table 7 show that socio-economic, governance, institutional factors, and their interplays significantly influence scheme farmers’ adaptive capacity. Adaptive capacity was significantly and positively related to HH_Age (p < 0.01), HH_Gender (p < 0.01), HH_Edu (p < 0.01), Tenur (p < 0.01), and Coop (p < 0.01). Irri_comm (p < 0.01), HHGender*Polic (p < 0.05), HH_Age*Credit (p < 0.05), and HH_Age*Tenure (p < 0.01) were significantly and negatively influencing adaptive capacity.

**Table 7:** Predictors of adaptive capacity.

	B	Std. Error	Beta
Intercept	0.317	0.074	
HH_Age	0.006***	0.001	0.889
HH_Gender	0.069**	0.027	0.366
HH_Edu	0.005**	0.002	0.164
Tenur	0.103**	0.045	0.439
Tradit	0.005	0.006	0.044
Coop	0.010***	0.004	0.141
Pvt_NGOs	-0.001	0.006	-0.014
Acade	0.010	0.006	0.093
Irri_comm	-0.018***	0.005	-0.175
Gvt_agenc	-0.003	0.009	-0.020
Polic	0.024	0.018	0.125
Credit	0.046	0.029	0.367
HHGender*Polic	-0.047**	0.020	-0.256
HH_Age*Credit	-0.001**	0.001	-0.544
HH_Age*Tenure	-0.002***	0.001	-0.640

Note: ***, ** and * indicate significance level at 1%, 5% and 10% respectively.

**Fig 1:**
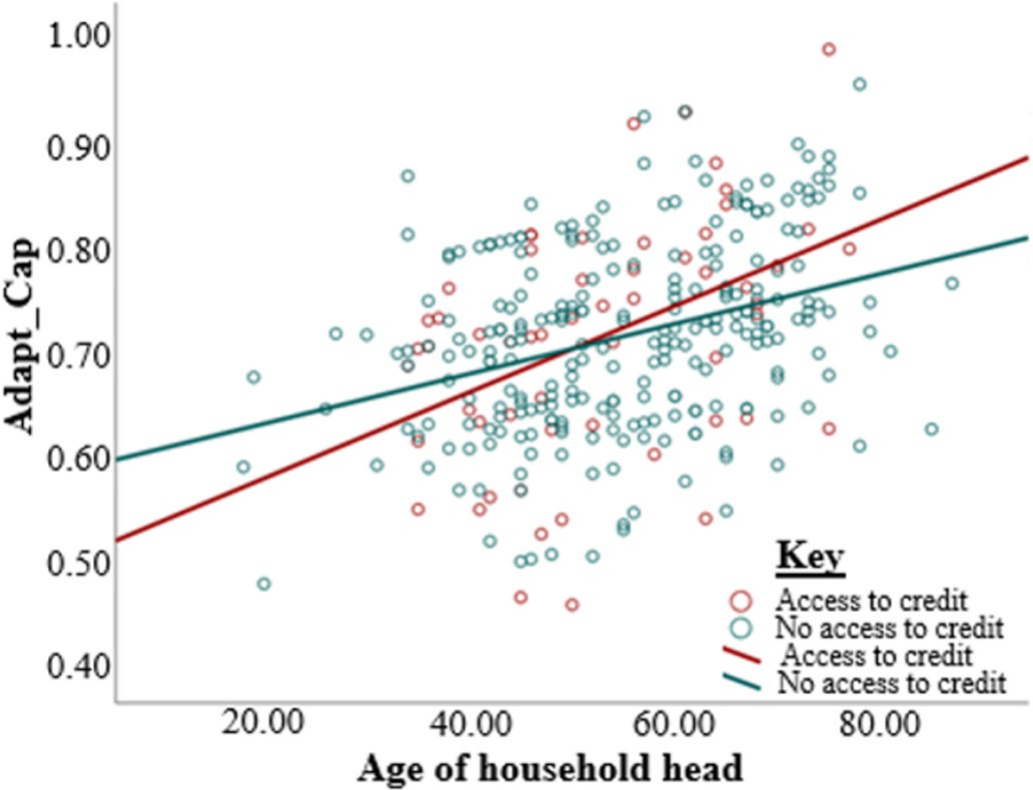
The interaction between the age of the household head and access to credit (all other factors controlled).

**Fig 2:**
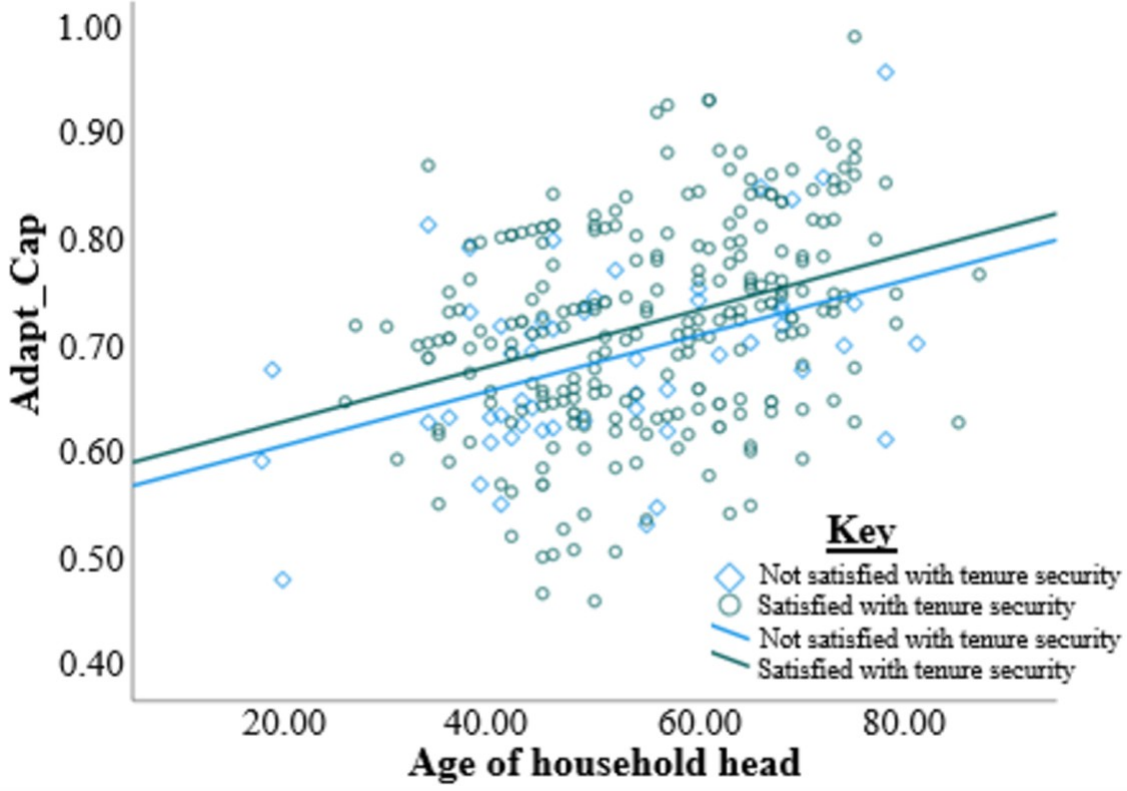
The interaction between the age of the household head and land tenure security (all other factors controlled).

**Fig 3:**
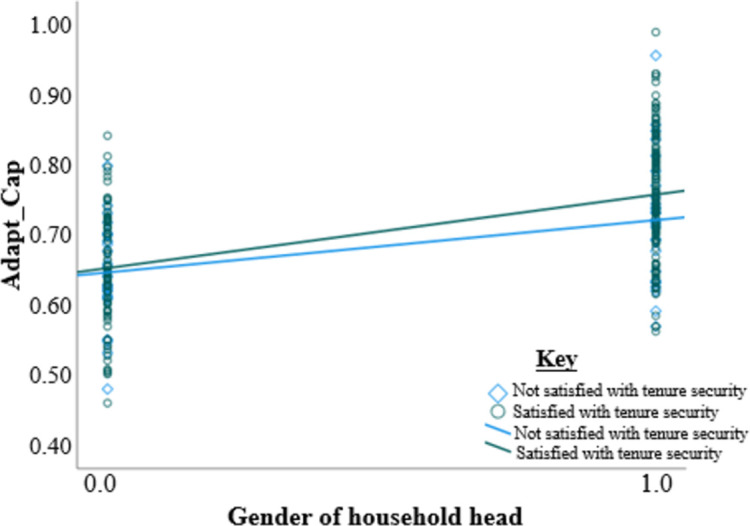
The interaction between gender and land tenure security (all other factors controlled).

In addition, two regression lines were drawn between age of household head and adaptive capacity was drawn according to access and no access to credit (Fig 1). Similarly, two regression lines were constructed between satisfaction and no satisfaction with land tenure security (Fig 2). The effect of access to credit on the contribution of age to adaptive capacity can be seen by the differences in gradients of the two lines Fig 1. The regression lines in Fig 2 show the moderation of land tenure security on the effect of the age of household head on adaptive capacity. Fig 3 shows the moderation effects of land tenure security on the relationship between gender and adaptive capacity.

## 4. Discussion

Our ex-ante expectations were that elderly farmers are more adaptive to climate change due to high farming experience (Table 8), making them good observers of environmental change and knowledgeable in adaptation actions to take [32]. As households head gets older, they become more adaptive to climate change. This study indicated that the relationship between age and adaptive to climate change was positive and weak (p < 0.01). Findings from this study support reports by previous studies that age positively relate to adaptive capacity [32, 75]. The results of this study were contrary to findings by Mwalukasa, Mlozi [76], who reported a negative impact of age on adaptive capacity. Further, Mwalukasa, Mlozi [76] suggest that although experience is higher for older farmers, age retards innovative adaptation due to the growing need for technological usage.

**Table 8:** Abbreviations and expected effect on adaptive capacity.

Abbreviation	Meaning	Expected effect
Ada_Cap	Adaptive capacity	
HH_Age	Age of the household head	+
HH_Gender	Gender of the household head	_+
HH_Edu	Years of formal education of the household head	+
Tenur	Household head satisfied with land tenure.	+
Tradit	Effectiveness of Traditional Leaders on irrigation farming	+
Coop	Effectiveness of cooperatives on irrigation farming	+
Pvt_NGOs	Effectiveness of private organizations and NGOs on irrigation farming	+
Acade	Effectiveness of academic institutions on irrigation farming	+
Irri_comm	Effectiveness of irrigation committees on irrigation farming	+
Gvt_agenc	Effectiveness of Traditional Leaders on irrigation farming	+
Polic	Awareness of government initiatives and policies on irrigation schemes	+
Credit	Access to credit	+

Similarly, the positive coefficient of gender means that males are more adaptive and female farmers have a limited probability of obtaining higher adaptive capacity (Table 6). Climate change is gendered in nature; hence, most of the responsibilities of women are climate-sensitive, making them suffer more because of climate variability than men [77]. Female farmer’s lower adaptive capacity is attributed to limited access to production inputs [68]. Women characteristically have limited access to productive resources (assets, inputs and services) compared to men [78–80], militating their adaptation to climate change and variability. Mosello, Oates [81] find out that female-headed households in Zimbabwe are particularly vulnerable since they hold less land, produce lower yields, own fewer heads of livestock and are excluded from access to services like extension and credit. Results obtained from the study further revealed that 53.8% of the female household heads were not married, while a majority of male household heads were married (89.5%). Women’s marital status is a factor in determining their access to adaptive strategies compared to the case of men [82]. Findings by Van Aelst and Holvoet [82] show that divorced and widowed women are disadvantaged in agricultural water management although they can pursue more income-earning activities outside the farming sector. A study in Mozambique suggests that female-headed households have a lower adaptive capacity [83].

The positive coefficient of education implies that education is key for reducing uncertainties and ensure sustainable agricultural practices, hence increasing the adaptive capacity of smallholder farmers in SISs. Education is one of the generic capacities usually associated with development policies [9]. Findings by Mosello and Oates [81] that low educational levels and lack of skills and weak leadership undermine farmers’ ability to manage their schemes effectively. More educated and experienced farmers have improved access to infrastructure and market, greater capacity to manage and analyse information and use it more efficiently [84, 85]. Educated farmers have a higher opportunity to improve their production, access information, and understand commercial farming concepts that are critical in adapting to climate change [86, 87]. Education may potentially positively influence the ability of the household to take advantage of risk management mechanisms, hence, improving the household’s overall adaptive capacity. These results are consistent with others who suggest that education improves adaptive capacity of households [84, 85, 88, 89]. A study by Asante and Boakye [90] revealed that education positively impacts households with low adaptive capacity.

In terms of land tenure, the results show that farmers satisfied with the land tenure security in the scheme were more likely to adapt to climate change (p < 0.05). The results from this study support the hypothesis that land tenure positively relates to adaptive capacity (Table 8). Farmers in the three schemes did not hold any title deeds to the irrigation land; hence the scheme management is responsible for allocating and transferring the scheme from one user to another. These results concur with Alemayehu and Bewket [32] finding that land tenure security affects the choice of adaptation strategies. Perceived land tenure security impacts land management activities, positively affecting adaptation options due to a higher propensity to engage in multiple adaptation options [32].

The positive relationship between adaptive capacity and satisfaction with cooperatives in the scheme illustrates the importance of collective action on adaptation. These findings imply that farmers’ access to support services that include credit, training and information from cooperatives is more likely to make them more adaptive to climate change. Farmers satisfied with cooperatives can potentially be members of cooperatives. Participating in cooperatives is potentially instrumental in shaping farmers’ motivation and facilitate the decision to adapt to climate change through cooperatives [91]. Cooperative plays a pivotal role by enabling technological adaptation by promoting certain technologies in Zimbabwe [92]. Cooperatives promote effective exchange and co-production of local and scientific information and provide new arenas for social interaction [91]. Members of cooperatives potentially use the best practices approach as they are likely to receive climate change training and better access to financial assistance [93]. The ability of individual actors to retain bonding enables members to gain the benefits of cooperatives.

The IMCs in SISs is responsible for conflict resolution, acquiring and managing funds, and maintaining and improving scheme infrastructure. The results from the study show that participants who were satisfied with the IMCs were less adaptive to climate change. A unit change in satisfaction result in reduced adaptive capacity by a margin of 0.25 at a 1% significant level (Table 6). The study shows that 86.2% of the participants were satisfied with the irrigation management committee. The study’s findings support observations in the Makwe irrigation scheme in Zimbabwe, where 85% of the farmers were satisfied with the irrigation management committee [94]. The negative relationship between satisfaction with IMCs and adaptive capacity implies that IMCs do not consider adaptation to climate change in their activities in water-related issues. The IMCs mobilizes government support, donor funding, financial management, mobilizes participation of members in scheme maintenance, ensures equitable distribution of water and information sharing, and facilitates the development of collective adaptation that is context-specific to the risk the scheme faces. Villamayor-Tomas and García-López [9] postulate that IMCs can provide area-specific adaptive to cope with water-related climate disturbances.

The critical findings show that awareness to irrigation policies significantly and negatively moderates the relationship between gender and adaptation to climate change. Farmers’ knowledge of irrigation policies can negatively impact the contribution of age to adaptive capacity. Although awareness of irrigation policies has no significant direct effect on adaptive capacity, its impact on adaptive capacity is moderating by gender. Previous studies show gender inequality in agricultural policies formulation and awareness, where females are distinctively more compromised [95, 96]. Policies are critical measures for adaptation purposes at local, national and international levels [97]. Hence limited attention of Zimbabwe’s agricultural policies to the impacts of climate change [97, 98] is of significant concern. Negative moderation of age effect on adaptation to climate change might relate to lack of favourable agricultural policies to climate change adaptation [97]. To get rid of the negative moderation of irrigation policies on adaptive capacity, the government of Zimbabwe needs to adopt favourable irrigation policies at the local and national levels.

The study finds out that the positive impact between the age of the household head and adaptation to climate change was significantly and negatively moderated by perception about land tenure security. Thus, perception of land tenure security can negatively impact the contribution of experience farmers attain as they grow older to adapt to climate change. Land tenure security and age of the household head were significantly and positively related to adaptive capacity; however, the negative mediation effect reflects the inverse relationship between the two factors. Hence, aging farmers were land tenure insecure than young farmers. Several studies reported diverse perceptions on land tenure security among smallholder irrigation schemes in Zimbabwe [99]. According to Makanyisa, Chemhuru [100], land tenure security is affected by socio-economic, political and sphere in Zimbabwe. The complex land tenure system among smallholder irrigation schemes across the nation implicates land tenure security [101], challenging adaptation to climate change with the advancement of age.

On the other hand, access to credit negatively moderated the positive relationship between age and adaptive capacity. Farmers’ access to credit can negatively impact the positive relationship between the age of household head and adaptive capacity. In Zimbabwe, access to credit influences farmers’ irrigation development participation [102]. Challenges of accessing credit in smallholder irrigation schemes were highlighted [86, 87, 103]. Farmers in smallholder irrigation schemes of Zimbabwe face challenges in receiving credit through formal financial institutions mainly due to lack of collateral security and lack of formal land tenure [86]. Credit and financial facilities are reported as key components of revitalizing irrigation schemes in Zimbabwe.

This study shows that the age of the household head has a unique relationship with access to credit, which concurs with the previous findings [86, 104, 105]. The regression lines in Fig 1 show that access to credit has a strong regression effect than lack of access to credit. The results show that age is particularly important for farmers to access credit. These results suggest that the effect of access to credit is more prominent for aging farmers. The interaction of access to credit and age of scheme farmers seems to have a far-fetched impact on adaptation to climate change. This suggests that access to credit plays a crucial role in aging farmers’ decisions on implementing adaptation strategies.

Further, the study shows the interaction between age and land tenure security as reported by previous studies [106–108]. Fig 2 shows a similar regression effect of land tenure security of the two regression lines. However, the freehold nature of land tenure in smallholder irrigation schemes in Zimbabwe makes aging farmers unable to access credit due to freehold land tenure unable to invest for climate change adaptation [109, 110]. The results from the study show that land tenure security negatively moderates the relationship between age and adaptive capacity. The findings from this study contradict the report by Zikhali [110]. Farmers attain perceived land tenure security every consecutive year that they occupy the parcel of land, which increases by the farmer’s age. Land tenure security increases with the consecutive increase in the age of the farmers.

On the other hand, land tenure security is closely linked to the gender of the household head. The relationship between land tenure security and gender of household heads in Zimbabwe’s agriculture context was highly emphasized [111–114]. Land tenure security is gendered, and women are mostly disadvantaged [113]. Fig 3 shows that the availability of land tenure security has stronger regression effects than the absence of land tenure security. The findings from this study reveal that land tenure security negatively affects females compared to their male counterparts. The moderation of gender by land tenure negatively impacts adaptation to climate change. Land tenure security helps farmers to have confidence in investing towards climate change adaptation [106]. However, the gender of the household head determines the esteemed land tenure security, such that when land tenure security is absent, gender can negatively impact adaptation to climate change.

Although adaptation to climate change is widely studied, there is limited research on the interaction of factors on adaptation. This study relates to the previous studies on adaptation but has overlapped on innovation. This study relates to previous studies that reported the effects of age, land tenure, gender, and access to credit on adaptation to climate change. The study confirms the relationship between the above factors and adaptive capacity in the smallholder farming sector. Further, based on vulnerability theory, such factors are key determinants of adaptation to climate change.

### 4.1 Implications

The current study raises essential implications on applying interactions to assess adaptation to climate change. Therefore, understanding the institutional, governance and socio-demographic interaction is crucial to improve adaptation in smallholder irrigation farming. Besides, this study shows that the link between age and adaptive capacity is moderated by two factors (access to credit and land tenure security. Specifically, access to credit and availability of land tenure security negatively impacts the link between age and adaptive capacity. This study strongly recommends understanding the conditions of land tenure security and access to credit that contribute to the negative impact of their interaction with age on adaptation to climate change. Further, land tenure security is of particular importance given that it has negatively moderated the link between age and gender and adaptive capacity. To improve farmers’ participation towards adaptation to climate change in scheme, there is a need to address the factors contributing to the negative impact of land tenure, access to credit, and awareness to policies to link age and gender with adaptive capacity. Climate change adaptation policies need to consider the interaction of factors.

### 4.2 Limitations

This study has some limitations. First, the dependent variable used an index calculated based on the weighted balance approach of which the factors may or may not carry the same weight. Second, this study was a questionnaire-based study among smallholder irrigation schemes which may make it difficult to convey feelings and emotions and may also have unconscientious responses. Thirdly, this study was conducted on a limited population in three schemes.

## 5. Conclusion

This study illustrates how socio-demographic, institutional and governance factors and their interactions influence the adaptive capacity of smallholder irrigation farmers in Zimbabwe. Notably, this study identifies that access to credit and land tenure security negatively and significantly modify the link between the age of the household head with adaptation capacity. Similarly, awareness of irrigation policies negatively and significantly modified the interaction between the gender of the household head with adaptive capacity. The findings from this study will contribute to the body of knowledge on adaptation to climate change and the impacts of the interaction of factors on the adaptive capacity of smallholder irrigation communities. Future studies on the interaction of factors are recommended at temporal and spatial scales to widen understanding of its contribution towards climate change adaptation among smallholder farming systems. Since higher adaptive capacity decreases livelihood vulnerability to climate change, understanding the interplay of factors and addressing it will help scheme farmers cope with anticipated extreme climate change and vulnerability cases. This study expands the knowledge of adaptation to climate change by discussing the interplay of factors.

## Supporting information

S1 File (DOCX)
