# Supplementary material for: Socio-demographic, institutional and governance factors influencing adaptive capacity of smallholder irrigators in Zimbabwe
Source: PLoS One. 2022 Aug 29;17(8):e0273648. doi: 10.1371/journal.pone.0273648 (PMC9423651; doi:10.1371/journal.pone.0273648)
Supplement: S1 File — (DOCX) [file pone.0273648.s001.docx]

**Questionnaire**

**University of KwaZulu-Natal**

**School of Agricultural, Earth and Environmental Sciences**

***Note:*** *The information captured in this questionnaire is strictly confidential and will be used for research purposes by staff and students at the University of KwaZulu-Natal.*

***TOPIC: The governance-institutions nexus in water management for climate change adaptation in smallholder irrigation schemes in Zimbabwe***

| Date |  | Enumerator |  |
| --- | --- | --- | --- |
| Household Code |  | Irrigation Scheme |  |
| Village |  |  |  |

*(Tick /circle correct answer where applicable)*

**A1: Socio-Demographic Characteristics**

|  | **Question** | | | | | **Response** | | | |
| --- | --- | --- | --- | --- | --- | --- | --- | --- | --- |
| *a* | Age of household head* | | | | |  | | | |
| *b* | Gender of household head | | *Male* |  | *Female* | | |  | |
| *c* | Marital Status of household head | *Single/Divorced/Widowed* | |  | *Married* | | |  | |
| *d* | Number of years the household head spent in formal education | | | | |  | | | |
| *e* | Main occupation of household head | *Fulltime farmer* | *Other (specify below)* | | | | | |  |
|  |  | | | | | | | | |
| *f* | Total number of household members | | | | |  | | | |
| *g* | Number of household members below 15 years | | | | |  | | | |
| *h* | Number of household members above 65 years | | | | |  | | | |
| *i* | Are there any children less than 18 years old living in your home because one or both parents are deceased/ not children of the family? | | | | | *Yes* | *No* | | |
| *j* | If yes in (7.i), how many are they? | | | | |  | | | |
| *k* | How many household members help with or involved in farming regularly? | | | | |  | | | |

*(*stays at home for 3 or more days per week.)*

**A2 Livelihood Strategies**

|  | **Question** | | | | **Response** | | |
| --- | --- | --- | --- | --- | --- | --- | --- |
| *a* | How many people in your family go to a different community to work? | | | |  | | |
| *b* | Do you or someone else in your household raise livestock? | | | |  | | |
| *c* | Do you or someone else in your household grow crops? | | | |  | | |
| *d* | Do you or someone in your household collect something from the bush, forest, lakes and rivers for sale? | | | |  | | |
| *e* | If yes in (d), can you name what they collect |  | | | | | |
| *f* | Do you fetch expected prices after harvesting your crops? | | *Yes* |  | | *No* |  |

**A3 Household Health**

|  | **Question** | | | **Response** | |
| --- | --- | --- | --- | --- | --- |
| *a* | How many hours does it take you to get to a health facility? | | |  | |
| *b* | Is anyone in your family chronically ill? (get sick very often) | | |  | |
| *c* | If yes in 2.i), how many? | | |  | |
| *d* | Has anyone in your family been so sick in the past 2 weeks that they had missed work or school? | | |  | |
| *f* | Did you reduce your expenditure on food during drought? | *Yes* |  | *No* |  |

**A4 Social Networks**

|  | **Question** | | | **Response** | |
| --- | --- | --- | --- | --- | --- |
| *a* | In the past month, did your relatives or friends help you and your family? | | |  | |
| *b* | Did you or your family help relatives and friends in the past month? | | |  | |
| *c* | Did you borrow any money from relatives/friends in the past month? | | |  | |
| *d* | Did you or anyone in your family lend money to relatives/friends in the past month? | | |  | |
| *e* | In the past 12 months, have your family get assistance from government NGO/? | | |  | |
| *f* | Is any of your household a member of any organisation / cooperatives? | *Yes* |  | *No* |  |
| *g* | Do you own a TV and / or Radio in your home? | *Yes* |  | *No* |  |
| *h* | Do you discuss your farm related problems with other farmers or extension workers? | *Yes* |  | *No* |  |

**A5 Food**

|  | **Question** | | | | | | **Response** |
| --- | --- | --- | --- | --- | --- | --- | --- |
| *a* | What are the main sources of your family food? | | | | *Own farm* | *Working family member* | |
|  | *relatives/friends* | *Remittances* | *relief* | *Others (Specify)* | | | |
| *b* | Does your family always have adequate food for the whole year? | | | | | |  |
| *c* | If **No** in 2b), how many months a year that your family have trouble getting food? | | | | | |  |
| *d* | Does your family save some of the crops you harvest to eat during a different time of the year? | | | | | |  |
| *e* | Does your family save seeds to grow the next year? | | | | | |  |
|  | How many hours does it take you to reach the market? | | | | | |  |

**A7 Natural Disasters and Climate Variability**

|  | **Question** | | | | | | **Response** | |
| --- | --- | --- | --- | --- | --- | --- | --- | --- |
| *a* | How many times has this place affected by drought between 2009 – 2019? | | | | | |  | |
| *b* | Did you receive early warning about droughts before they occurred? | | | | | |  | |
| *c* | Did anyone in your family die due to drought in the past 10 years? | | | | | |  | |
| *d* | Is anyone in your family trained to deal with floods /drought/ cyclones? | | | | | |  | |
| *e* | Did occurrence of drought increase in the last 10 years? | | | | | | *Yes* | *No* |
| *f* | Do you think that drought will increase in the future? | | | | | | *Yes* | *No* |
| *g* | Did you grow different crop varieties in the last 10 years | | | | | | *Yes* | *No* |
|  | What are the indicators of a changing climate in your region? | | | | | *Shortened rainy season* | | |
|  | *Long dry spells* | *Erratic rainfall* | | *Late rainfall* | *Increased frequency of droughts* | | | |
|  | *Shortened cold season* | | *Others (Specify)* | | | | | |

**A8 Farming Experience**

|  | **Question** | **Response** |
| --- | --- | --- |
|  |  |  |
| *a* | Number of years farmers have been into farming? |  |
| *b* | Number of years farmers have been into irrigation farming? |  |

**A9 Water Availability and Irrigation**

**1. May you please complete the table below on availability of irrigation water?**

|  | **Question** | | | | | | | | | | | | | | | | | **Response** | | | | | | |
| --- | --- | --- | --- | --- | --- | --- | --- | --- | --- | --- | --- | --- | --- | --- | --- | --- | --- | --- | --- | --- | --- | --- | --- | --- |
| *a* | How many hours do you take to get to water source? | | | | | | | | | | | | | | | | |  | | | | | | |
| *b* | What is your position along the primary canal? | | | | | | | | *Head* | |  | | | *Middle* | | |  | | | *Tail* | | | |  |
| *c* | Is water discharged in time? *[1-Yes, 0-No]* | | | | | | | | | | | | | | | | |  | | | | | | |
| *d* | On average how many days per week do you irrigate your crops? (*indicate number*) | | | | | | | | | | | | | | | | |  | | | | | | |
| *f* | How do you know when your crops need to be irrigated? | | | | | | *irrigate when it’s my turn* | | | | | | | | | | | | | | | |  | |
|  | *when the soil is dry* |  | *when crops are stressed* | | |  |  | | | | | | | | | | | | | | | | | |
| *g* | When was the problem of water shortage last experienced? | | | | | | | *it is currently there* | | | | | | | | | | | | | | | |  |
|  | *a few days ago* | | |  | *a few weeks ago* | | | |  | *-a few months ago.]* | | | | | | | | |  | | | | | |
| *h* | Do you think that there will be water scarcity in the coming years? | | | | | | | | | | | *Yes* | | | |  | | *No* | | |  | | | |
| *j* | Water quality has decreased over the past 10 years | | | | | | | | | | | | *Yes* | |  | | | *No* | | | |  | | |

**SECTION B: INFRASTRUCTURE**

1. What is your level of satisfied with the state of the following infrastructure?

*KEY:1=Strongly dissatisfied 2=Dissatisfied 3=Neutral 4=Satisfied 5=Strongly satisfied*

| **Infrastructure** | **State level of satisfaction** |
| --- | --- |
| a. Road accessibility |  |
| b. Markets |  |
| c. Agricultural water supply |  |
| d. Drinking water supply |  |
| e. Others (specify) |  |
|  |  |

**SECTION C: FARMING IMPLEMENTS**

1. Do you own the following assets? *(Indicates number and value of assets owned,).*

| **Farm assets** | \| **1-1-Yes 0-No** \| \| --- \| | **Number of working assets** | **Their estimated value** |
| --- | --- | --- | --- | --- |
| Oxcart |  |  |  |
| Ox-drawn plough |  |  |  |
| Cultivator |  |  |  |
| Hoes |  |  |  |
| Knapsack Sprayer |  |  |  |
| Wheelbarrow |  |  |  |
| Others (Specify) |  |  |  |

**SECTION D: LIVESTOCK OWNERSHIP**

| **Livestock** | \| **1-1-Yes 0-No** \| \| --- \| | **Number owned** |
| --- | --- | --- | --- |
| Cattle |  |  |
| Goats |  |  |
| Chicken |  |  |
| Others |  |  |
|  |  |  |

**SECTION E: LAND HOLDING AND UTILISATION**

1. What is the total area of land your household owns/operates?

| **Type of farming** | **Ownership** | **Size of Land in Ha** | **Land fee paid per Year in ZAR** | **Size of cultivated land** |
| --- | --- | --- | --- | --- |
| Irrigation | Owned |  |  |  |
|  | Rented |  |  |  |
|  | Leased |  |  |  |
| Dry land | Owned |  |  |  |

**SECTION I: LAND TENURE**

1. Are you satisfied with the tenure security of your plots? *[1-Yes, 0-No]*
2. Are you permitted to sell your plots? *[1-Yes, 0-No]*

**SECTION K. SCHEME INSTITUTIONS**

1. **Please rank the following institution on their effectiveness on irrigation farming and state their roles.**

***Ranking Key:*** *1-* *Strongly Disagree, 2 - Disagree, 3 - Neutral, 4 – Agree, 5 – Strongly Agree*

***Function Key****:1-water allocation, 2-Input/Output markets, 3-Extension services/training, 4-water allocation and distribution, 5-Maintenance of irrigation scheme, 6-Supply inputs, 7-legal, 8-water charging, 9-financial management 10-Others (Specify)*

| Institution | Rank |
| --- | --- |
| Traditional Leaders |  |
| Cooperatives |  |
| Private Organisation and NGOs |  |
| Academic Institutions |  |
| Irrigation Committee |  |
| Community/fellow farmers |  |
| Government agencies (Extension Officer) |  |

**SECTION L. FARMERS’ PARTICIPATION IN IRRIGATION WATER MANAGEMENT**

1: Are there any irrigation committee in your area? *[1-Yes, 0-No]*

*2. How much money do you contribute for scheme maintenance per year? _______________*

*3.* Are you aware of government initiatives and policies on irrigation schemes? *[1-Yes, 0-No] __*

*4. Rank your participation (0-never, 1-sometimes, 2-always)*

| Institution | Rank |
| --- | --- |
| Attend irrigation meetings |  |
| Electing/removing committee members |  |
| Formulating scheme rules |  |
| Irrigation Water scheduling |  |
| Scheme maintenance |  |
| Contribute finance |  |
| Attending irrigation meetings |  |
| Attending water related training |  |
| Help other farmers to manage/conserve water |  |
| Engage authorities regarding water issues in the area |  |

**SECTION N. WATER AVAILABILITY AND IRRIGATION**

**Rank the following statements in relation to water access and availability**

1. Strongly Disagree, 2 - Disagree, 3 - Neutral, 4 – Agree, 5 – Strongly Agree

|  | **Questions** | **Rank** | | | | |
| --- | --- | --- | --- | --- | --- | --- |
|  |  | **1** | **2** | **3** | **4** | **5** |
| *a* | Water source reliable (never dries up) |  |  |  |  |  |
| *b* | Water is sufficient for my cropping requirements |  |  |  |  |  |
| *c* | I am satisfied with the maintenance of the canal |  |  |  |  |  |
| *d* | Water distribution/sharing at farm level is fair |  |  |  |  |  |
| *e* | Water supply to my plot is reliable |  |  |  |  |  |
| *f* | Conflict resolution mechanisms are effective |  |  |  |  |  |
| *g* | The penalty system for non-compliance is effective |  |  |  |  |  |
| *h* | I often participate in infrastructure maintenance |  |  |  |  |  |
| *i* | Availability of water has decreased over the last 10 years |  |  |  |  |  |

**SECTION O. PEST AND DISEASE VULNERABILITY**

|  | Which insects of your crops have increased in past 10 years? | Which crops are affected? |
| --- | --- | --- |
| *a* | *Aphids* |  |
| *b* | *Cutworms* |  |
| *c* | *Armyworms* |  |
| *d* | *Red spider mite* |  |
| *e* | *Beetles* |  |
| *f* | *Bollworms* |  |
| *g* | *Whiteflies* |  |
| *h* | *Termites* |  |
| *i* | *Others (Specify)* |  |

| **May you please complete this section on insect pests** | | | | | | | | | | | | | | | | | | | | | | | |
| --- | --- | --- | --- | --- | --- | --- | --- | --- | --- | --- | --- | --- | --- | --- | --- | --- | --- | --- | --- | --- | --- | --- | --- |
| *a* | If there is an increase in number of insect pests, what factors do you think have led to | | | | | | | | | | | | | | | | | | | | | | |
|  | increased incidence of these insect pests? | | | | | | | | | | | | *Shortened winters* | | | | *Warmer temperatures* | | | | | | |
|  | *Increased dry spells* | | | | | | | *Insect pests resistance* | | | | | | | *Poor insect pest management* | | | | | | | | |
|  | *Low rainfall* | | | | *Others (Specify)* | | | | | | | | | | | | | | | | | | |
| *b* | Which season of the year are these insect pests most prevalent? | | | | | | | | | | | | | | | | | *Summer* | | | | *Autumn* | |
|  | *Winter* | *Spring* | | | | | *All Year round* | | | | |  | | | | | | | | | | | |
| *c* | How do you view the extent of crop losses from insect pest and disease attack? | | | | | | | | | | | | | | | | | | | | | | |
|  | *It is high* | | *It is moderate* | | | | | | *It is low* | | | | | | | | | | | | | | |
| *d* | Which major strategy do you use to control insect pests? | | | | | | | | | | | | | | | *Insecticides* | | | | | *Biological* | | |
|  | *Cultural* | | | *Integrated Insect Pest management* | | | | | | | | | | | | | | | | | | | |
| *e* | Are the chemical insecticides effective in the control of the insect pests? | | | | | | | | | | | | | | | | | | | *Yes* | | | *No* |
| *f* | If No, give reasons to your answer on question | | | | | | | | | | | | | | | | | | | | | | |
|  |  | | | | | | | | | | | | | | | | | | | | | | |
| *g* | How frequent do you spray these chemical insecticides? | | | | | | | | | | | | | | | *Once/month* | | | *Twice /month* | | | | |
|  | *Once /week* | | | | | *Twice /week* | | | | *Other, (specify)* | | | | | | | | | | | | | |
| *h* | Did you change your spraying frequency from what you used to do last year? | | | | | | | | | | | | | | | | | | | | | | |
|  | *Yes increase* | | | | | *Yes, decrease* | | | | | *No* | | |  | | | | | | | | | |

**SECTION P: CROPPING AND MARKETING**

1. **Fill the table about the market you sell your main crop**

Please indicate the crops you planted in the past summer season, the area you planted, the output you produced and the costs you incurred

| **Crop** | **area planted** | **Quantity harvested (kgs)** | **Quantity sold** | **Inputs used** | **Quantity purchased** | **Cost per unit** |
| --- | --- | --- | --- | --- | --- | --- |
| **Summer 2019/20 season** | | | | | | |
|  |  |  |  |  |  |  |
|  |  |  |  |  |  |  |
|  |  |  |  |  |  |  |
| **Winter 2020 season** | | | | | | |
|  |  |  |  |  |  |  |
|  |  |  |  |  |  |  |
|  |  |  |  |  |  |  |
